# Evaluation of Gastric Lesions Based on *Helicobacter pylori* and *Helicobacter*-Like Organisms (HLOs) in Cats; A Histopathological and Bacteriological Study

**DOI:** 10.5812/jjm.9129

**Published:** 2014-06-01

**Authors:** Farhang Sasani, Javad Javanbakht, Farrokh Reza Kabir, Mehdi Agha Mohammad Hassan, Ali Reza Pashaei

**Affiliations:** 1 Department of Pathology, Faculty of Veterinary Medicine, Tehran University, Tehran, IR Iran; 2 Department of Clinical Science, Science and Research Branch, Islamic Azad University, Tehran, IR Iran; 3 Department of Clinical Science, Faculty of Veterinary Medicine, Tehran University, Tehran, IR Iran; 4 Private Veterinary Practitioner, Faculty of Veterinary Medicine, Tehran University, Tehran, IR Iran

**Keywords:** *Helicobacter pylori*, Cat Diseases, Mucous Membrane

## Abstract

**Background:**

The lesions induced by *Helicobacter pylori* in a candidate animal model should always be examined thoroughly. The resemblance of these lesions to those observed in humans can indicate whether the usage of this model will contribute to the understanding of the various pathogenic mechanisms involved in the development of human *H. pylori*-associated diseases.

**Objectives:**

The aim of this study was to perform a histopathological and bacteriological evaluation of gastric lesions based on *H. pylori* and *Helicobacter*-like organisms (HLOs) in cats.

**Materials and Methods:**

The present study was carried out on 28 cat’s (13 male and 15 female cases) gastric mucosae, which were tested by bacteriological and histopathological methods. Biochemical tests such as catalase, oxidase and urease were utilized in addition to Gram and Giemsa staining.

**Results:**

This research demonstrated that solely one case of *H. pylori *was isolated by gastric mucosal culture. Microscopically, the infected stomachs by HLOs comprised a mild to severe diffuse lymphoplasmacytic infiltration into the subglandular and gastric mucosa. Lymphoid follicles were also marked, particularly within pyloric tissues and mostly in displaced mucosal glands. For 75% of the gastritis cases, both HLOs and rapid urease tests were positive, whereas 83% of cases were more than one-year-old with gastritis. Furthermore, 75% of cats indicated gastritis, though 25% encompassed no gastritis; hence 20% had negative results for the rapid urease test and 25% for the Giemsa staining test. Such results may indicate that cats without gastritis were considered as free of HLOs pathogenic bacteria.

**Conclusions:**

These results suggest that most cases of gastritis were located in the antral region. Additionally, the isolation of *H. pylori *from domestic cats raises the possibility of zoonotic characteristics for the slightly pathogen; therefore transmission occurs from cats to human and vice versa.

## 1. Background

Recent studies suggest a high prevalence of gastric *Helicobacter* infection in cats. Gastric *Helicobacter*-like organisms (HLO) have been observed in gastric biopsies of 41% to 100% of clinically healthy (1-3), and 57% to 100% of vomiting cats (3, 4). The development of animal models is important for the study of *Helicobacter pylori* pathogenesis. The need for suitable animals for this purpose has been clearly stated (5). To date, persistent colonization of the gastric mucosa and infection with *H. pylori* has been induced experimentally in gnotobiotic piglets, (6, 7) barrier-born pigs, (7) nonhuman primates, (8, 9) gnotobiotic dogs, (10) conventional dogs, (10) specific-pathogen-free (SPF) cats, (18 mice (SPF, germ-free, athymic, and transgenic), (11, 12) SPF Mongolian gerbils, (13) rats, (14) and guinea pigs (15, 16). 

Spiral organisms have been described in the gastric mucosae of cats and dogs since the 19th century and are considered common inhabitants of the gastric mucosal niche (17). Three different morphological types were identified after ultrastructural analyses of the bacteria *in situ*; these types were originally presumed to represent various stages in the movement of one organism (18). Recent polyphasic taxonomy studies involving various isolates, however, revealed at least three different species belonging to the genus *Helicobacter*, namely, *H. felis *(13), *H. bizzozeronii *(6) and *H. salomonis *(11); these species are both phenotypically and phylogenetically highly related (19). These tightly coiled organisms were largely ignored by the scientific community until the isolation of *H. pylori *from the human gastric mucosa renewed interest in gastric bacteria (20, 21).

## 2. Objectives

The purposes of this study were evaluation of histopathology of gastric mucosal changes and bacteriology studies of gastric bacteria, bacterial culture and identification of the isolated organisms on the basis of morphology, urease and catalase tests in cats. Finally, determination of whether naturally occurring gastric bacteria were associated with cats' gastritis.

## 3. Materials and Methods

### 3.1. Animals, Preparation of Extracts, Isolation and Identification of Compounds

Gastric tissues were collected during necropsy from 28 young and old stray cats (13 cases (46%) were male and 15 (54%) were females) and blood samples were taken in EDTA solution. Small pieces of gastric mucosa from body and antrum of the stomach were imprinted on glass slides, and were then stained by the Giemsa method. Furthermore, mucosal sections from the body and antrum were placed in tubes containing urea agar. Subsequently, the tubes were incubated at room temperature, monitored hourly for up to eight hours and then assessed. Development of a pink color in the gel was considered as a positive result. For the purpose of gastric organisms’ isolation, small pieces of mucosal samples approximately 3 × 3 mm from antrum, body, fundus, and cardia zones of the stomach were gathered for the bacterial culture. Every sample was placed in sterile Brucella broth (Rimmel) or Thiogluconate, for transport, abated in a sterile grinder, and then inoculated into the culture medium (Code no. BR56; Oxoid). 

A Brucella agar base was supplemented with 10% horse blood and 5 mg/L trimethoprim, 10 mg/L vancomycin and 2500 µg polymyxin-B for initial isolation of *H. pylori *(15). All culture plates were incubated for 5 to 7 days at 37-42°C in a moist microaerophilic atmosphere (37°C, 10% C_2_, 5% O_2_, 85% N_2_). Furthermore, the cultured organisms were Gram stained in order to detect their staining adjectives and morphology. A loopful of culture was placed in two drops of urease reagent for the urease test, described previously (19). Standard methods were used for the catalase and oxidase tests. (Table 1).

**Table 1 .** Histopathological, Biochemical and Bacteriological Findings in Cats ^a^

-	Gastritis	Rapid Urease Test	Geimsa Staining	Gram Staining	*H. pylori* Culture
**Positive**	21 (75)	20 (80)	18 (75)	16 (94)	1 (5)
**Negative**	7 (25)	5 (20)	6 (25)	1 (6)	20 (95)
**Total**	28 (100)	25 (100)	24 (100)	17 (100)	21 (100)

^a^ Data are presented as No. (%).

### 3.2. Histopathological Evaluation

Every stomach was incised along the lesser curvature and the tissue samples were placed in 10% buffered formalin, and the sections of gastric tissues from the cardia, fundus, body and pyloric antrum of each cat were embedded in paraffin, prepared as 5 micron-thickness slices and stained with Hematoxylin and eosin and Giemsa methods (2.5% in cacodylate buffer (pH7)). A gastric score was assigned to each section as follows: 0 (normal), 0 to 10 lymphocytes or plasma cells per 400 field; with no lymphoid aggregates and a normal gastric epithelium, 1 (mild gastritis); 10 to 50 lymphocytes or plasma cells per 400 field; with less than two follicles per 20 field and a normal epithelium, 2 (moderate gastritis); 10 to 50 or more lymphocytes or plasmacells per 400 field; with two or more follicles per 20 field and 3 (severe gastritis); 10 to 50 or more lymphocytes or plasmacells per 400 field and marked epithelium alterations. The frequency of lymphoid follicles present per 20 field was also noted. The histopathological findings for cats infected with *Helicobacter* spp. were compared with cats without HLOs pathogens. Cats were considered as infected with *Helicobacter* spp. while two or three tests (rapid urease test, Giemsa staining and culturing) were positive based on Neiger et al. (2) reports.

## 4. Results

**Figure 1.**
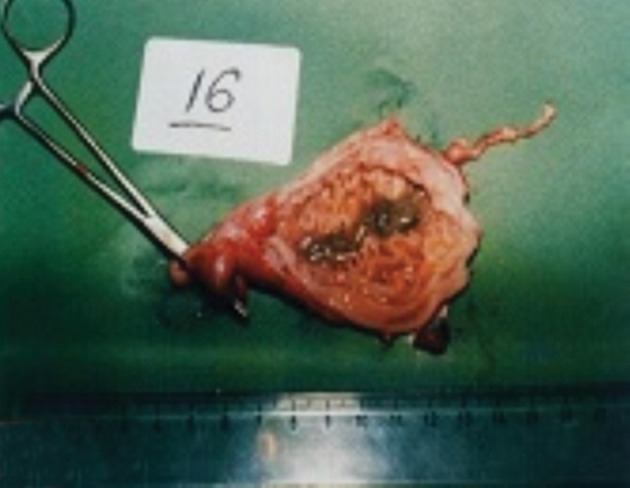
Lymphocytic Plasmacytic Gastritis in the Gastric Mucosa of a Cat With Naturally Acquired *Helicobacter* Species Infection Together with Chronic Follicular Gastritis (H&E Stain)

**Figure 2.**
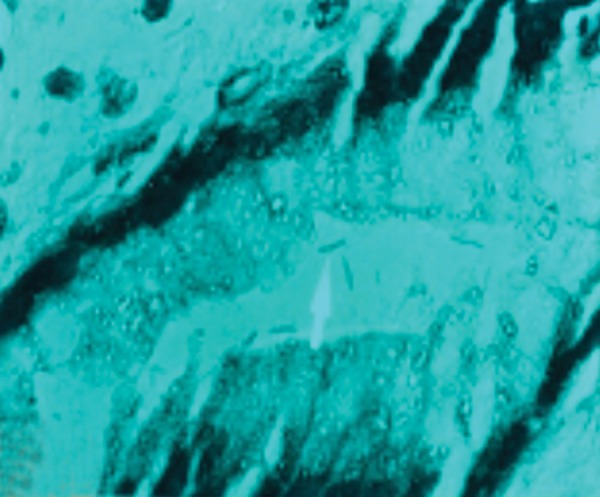
Arrows Indicate *Helicobacter* Species With a Spiral Shape and Dark Brown Colour in Cardia

**Figure 3.**
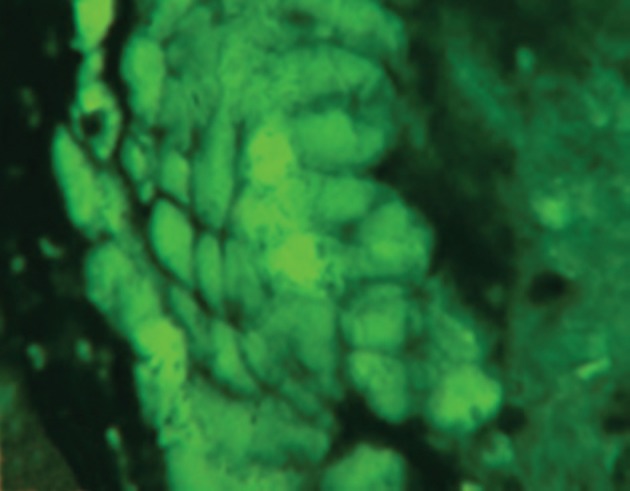
Photomicrograph Showing *H. pylori* in Gastric pit (Geimsa Staining, X1000, Grade)

**Figure 4.**
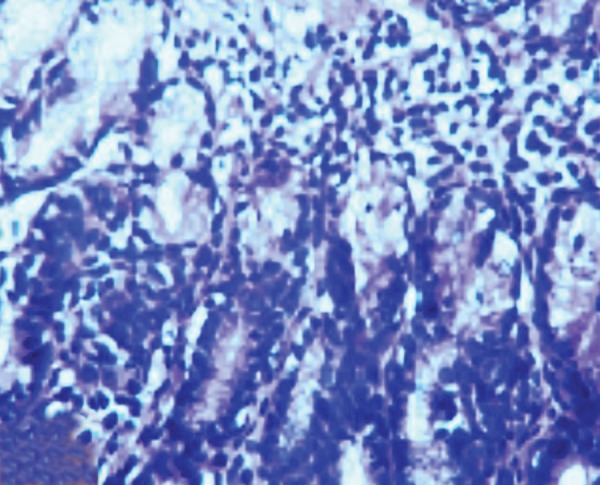
Lymphocytic Plasmacytic Gastritis in the Gastric Mucosa of a Cat with Naturally Acquired *Helicobacter* Species Infection Together with Chronic Follicular Gastritis (H&E Stain)

**Figure 5.**
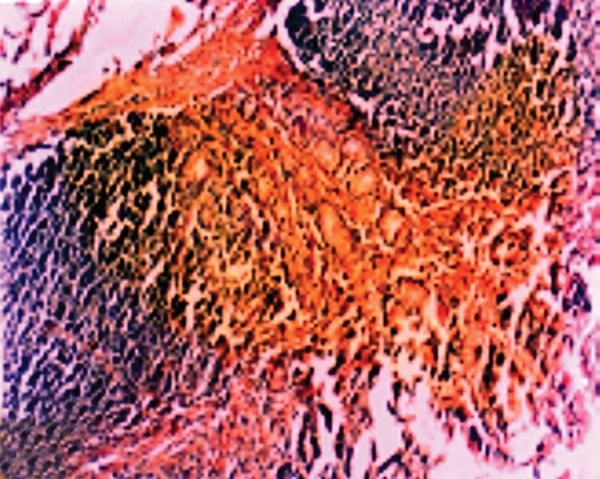
Mucosal Gastritis with Trichobezoar and *Toxocara Cati*

In histopathological analysis of gastric tissues from cardia, fundus, body and antral regions of 28 cases, 75% of cats were affected by gastritis without clinical signs (Figure 1). The antral region was mostly affected (78%) and the cardiac region (60%) was the least affected region by gastritis (Table 2). Following impression smears, in 94% of cases the cited bacteria were recorded by Gram staining. Concerning isolation of gastric spiral organisms, *H. pylori *was isolated from one cat but there were no possibilities for isolation of other HLOs spiral bacteria (Figure 2), (Figure 3), (Figure 4). Other bacteria such as pseudomonas aeroginosa, Bacillus cereus, *Escherichia. coli*, *Enterococcus fecalis*, *Bacillus subtilis*, *Staphylococcus epidermis *and *Proteus mirabilis* were isolated as well. Moreover, gastritis was observed (83%) in more than one-year-old cats. Leukocytosis was detected in 30% of cats with gastritis; however, 71% and 53% of cats developed neutrophilia and lymphopenia, respectively. Intestinal infestation of *Toxacara cati* (56% of cats) was also determined (Figure 5).

**Table 2.** Histopathological Finding of Gastric Tissues in Cats ^a^

-	Cardia	Body	Fundus	Antrum
**Positive gastritis**	15 (60)	16 (70)	19 (70)	18 (78)
**Negative gastritis**	10 (40)	7 (30)	8 (30)	5 (22)
**Total**	25 (100)	23 (100)	27 (100)	23 (100)

^a^ Data are presented as No. (%).

## 5. Discussion

The aim of the present research was to record the frequency of spiral organisms of *H. pylori *and to determine the severity and location of gastritis in cats. Herman et al. (4) and Neiger et al. (2) indicated that 80% and 75% of cases had positive results for *Helicobacter* spp. through rapid urease test and Giemsa staining test, respectively. The results of the two studies represented that 78% of 32 cats and 76% of 127 cats possessed HLO infection (4, 22). By implementing histopathological techniques, *Helicobacter* spp. has been identified in 82% of cats; infected and uninfected cats either showed nil or moderate to severe signs of a gastritis. Furthermore, no correlation between inflammation and infection has been detectable as defined by Brandenburg et al. (23) who claimed that the prevalence of gastric *H. pylori *infection in dogs and cats was 85-100% and *H. pylori *might be pathogenic in cats, whereas these species have been merely found in colony and not in domestic cats (24). 

In this study, *H. pylori *were isolated only from one cat from 20 tested stray cats. *H. pylori *appeared to be most prevalent in the antrum though in many animals plenty of such germs were detected in the fundus and body zones as reported by Handt et al. (25). Furthermore, Handt et al. explained that *H. pylori *presence in gastric tissue strongly supports the casual role of the bacterium in gastritis development (25). The multifocal lymphoplasmacytic infiltrate which often forms lymphoid follicles in *H. pylori *infected cats has been described for natural or experimental gastric *Helicobacter* infection in cats, dogs and ferrets as expressed by Handt et al. (25). There seems to be differences in the severity of gastritis in cats infected with diverse *Helicobacter* spp, as indicated by Neiger et al and Scanziani et al (26, 27).

In the present study, gastritis was observed in 75% of 28 cats while 25% of cases had no gastritis, so that 20% and 25% of them were negative in rapid urease test and Giemsa staining, respectively (Table 2). The obtained results may portend that cats without gastritis were free of *Helicobacter* spp. in their stomach. However, Hermanns et al. (4) reported that a relationship between the degree of colonization of HLOs and the extent of histopathological changes could be discovered in cats (28). In some studies (12-16), infection by *H. pylori*, *H. feli*s and *H. heilmannii*, has been associated with a moderate to severe lymphofollicular gastritis in 21 (88%) of 24 cats, and gastritis has markedly emerged in the antral region and consisted mainly of multifocal lymphoplasmacytic follicular infiltrates in deep mucosa due to chronic antigenic stimulation. In this study *Helicobacter* spp. have been abundant in fundus and most cases of gastritis were located in the antral region (78%) as well as fundus (70%), body (70%) and cardia (60%), which were involved with gastritis (Table 1). Due to the discovery of cats harboring *H. pylori* in a research colony (25) as well as in China (27) and according to preliminary data from France (29), cats may be a potential natural reservoir of *H. pylori* and could pose a zoonotic risk. In epidemiological studies, *H. pylori*-positive farm workers indicated greater contact with cats than with other animals (30). However, two studies evaluating *H. pylori* antibodies in cat owners and comparing them to humans without contact to cats revealed no increased risk in the first population (31, 32). A preliminary study on veterinarians had equally negative results for an increased risk of acquiring *H. pylori* infection from pets (22). Finally, isolation of *H. pylori* from stray and pet cats has not been possible for various studies (33, 34) suggesting that *H. pylori* infection in cats may be an anthroponosis, an animal infection with a human pathogen (9).

 The discovery of *H. pylori* on the surface water has shifted the possibility of direct transmission from pets even further (25). Several reports on human patients have assumed a possible zoonotic transmission of large GHLO from dogs or cats (35-37). Only recently, an identical ‘‘*H. heilmannii*’’ organism identified by PCR and urease-B gene sequencing has been found in a patient and one of his cats (38). Several case reports on GHLOs infection in human have suggested animals as a possible source of infection (17, 23). One epidemiological study supported the hypothesis that cats should be considered as a source of zoonotic spread of GHLOs; however, data focused on *H. felis *and *H. heilmannii*, not *H. pylori* (9).

It seems that the stray cat can be used as an experimental model in future investigations of *H. pylori*-induced pathogenesis as well as evaluation of anti-*H. pylori* prevention and treatment regimens (39). The possible risk of transmission of large GHLO to human patients is rather small, considering the greater than 90% prevalence in dogs and cats and the rare (0.5%) occurrence in humans. Notwithstanding, proper hygienic control is necessary to keep the risk at a minimum level.
